# Characterization of multitype colonies originating from porcine blastocysts produced *in vitro*

**DOI:** 10.3389/fcell.2022.918222

**Published:** 2022-09-12

**Authors:** Jong-Nam Oh, Jinsol Jeong, Mingyun Lee, Gyung Cheol Choe, Dong-Kyung Lee, Kwang-Hwan Choi, Seung-Hun Kim, Chang-Kyu Lee

**Affiliations:** ^1^ Department of Agricultural Biotechnology, Animal Biotechnology Major, and Research Institute of Agriculture and Life Sciences, Seoul National University, Seoul, South Korea; ^2^ Designed Animal and Transplantation Research Institute (DATRI), Institute of Green Bio Science and Technology, Seoul National University, Pyeongchang, South Korea

**Keywords:** embryo, lineage, segregation, RNA-seq, blastocyst attachment

## Abstract

Many types of embryonic stem cells have been induced from pre-implantation blastocysts to study the specification of early lineages. Various cell lines have been established using chemicals, including excessive inhibitory molecules. Previous studies have also aimed to purify cell populations representing a single embryonic lineage from a protocol. In this study, we used a novel culture condition to induce cells from blastocyst seeding and analyzed their characteristics. Next, signaling inhibitors were introduced during the cell culture period. Furthermore, we investigated the cell types using RNA sequencing. Each type of cell population showed a distinct morphology and reactivity with alkaline phosphatase. Marker proteins enabled each cell type to be distinguished by immunocytochemistry, and genes such as Sox17, Gata4, Gata6, T, and Cdx2 showed applicability for the discrimination of cell types. Signaling inhibitors suppressed the production of some cell types, and gene expression and marker protein patterns were collapsed. RNA-sequencing suggested cell-type-specific marker genes and the correlation among samples. In conclusion, four types of cells could be induced from porcine embryos using a single protocol, and they could be isolated manually. Our data will help promote the study of lineage segregation based on embryonic cells.

## Introduction

Every cell that comprises the body comes from a fertilized egg. From a few totipotent cells, embryonic lineages arise in a planned order. Following the compaction of embryonic cells, the outer cells form the trophectoderm (TE) and the inner cells form the inner call mass (ICM) (Yamanaka et al., 2006). Furthermore, cells in the ICM are segregated into epiblasts and primitive endoderm (PrE) (Hermitte and Chazaud, 2014). All of the cell populations of the fetus originate from these embryonic lineages (Rossant, 2007). To understand the early development of embryos, lineages in the early stages have been analyzed for various species (Teruel et al., 2000). However, some details remain unknown in mice, and even the principles of major specifications are unclear in model animals, including pigs.

To determine the mechanisms of lineage specification, many studies have tried to establish lineage-specific cell lines (Ralston and Rossant, 2005). Because embryonic events are rapid and the states of cells are constantly changing, maintenance of cellular conditions using chemicals is necessary (Teruel et al., 2000; Smith, 2001). Various embryonic stem cells representing specific lineages have been utilized to investigate the characteristics of each lineage.

In a previous study, we obtained an epiblast-like cell line from blastocyst seeding (Choi et al., 2019). Fetal bovine serum was excluded to reduce unknown factors, and many cytokines and chemicals were used to derive cells of the epiblast lineage (van der Valk et al., 2018). Inhibitors for the maintenance of stem cells were introduced to suppress unintended cell types. Many other studies have used various combinations of chemicals to lead cells on a path to a single lineage (Emre et al., 2007; Li and Ding, 2010). However, during the early stages of natural development, most signals originate from inside the embryo. Therefore, to follow the spontaneous development of early embryos, chemical barriers should be excluded. However, mimicry of the early differentiation of embryonic cell lineages outside of the reproductive tract still has challenges to overcome. The primary task is the isolation of cell populations representing embryonic lineages with the minimum use of inhibitors.

In this study, we minimized inhibitors to retain as many embryonic lineages as possible. Two inhibitors that are required to maintain pluripotent states in pigs, human leukemia inhibitory factor (hLIF) and basic fibroblast growth factor (bFGF), were used together under previously described culture conditions (Ezashi et al., 2012). We established four types of cells from *in vitro* fertilized blastocysts. To identify these cell types, first, the morphology of the cells was examined. Next, alkaline phosphatase staining and immunocytochemistry of marker proteins were conducted. Then, we isolated each cell type and used quantitative PCR to analyze the expression levels of marker genes. To identify detailed expression profiles, RNA sequencing (RNA-seq) was conducted with cell clumps. During the culture period, and to observe the response of cells, we also added additional signaling inhibitors (PDGFRA inhibitor, TGFβ inhibitor, and MEK inhibitor), which have the potential to control the pluripotency of pig embryos (Vrbsky et al., 2015; Oh et al., 2020). Finally, we isolated RNAs from each cell type and conducted RNA-seq. We selected upregulated genes from each cell type and showed the distribution of each type on a multidimensional scaling plot. Additionally, the results were compared with sequencing results from other studies.

## Materials and methods

The care and experimental use of pigs were approved by the Institute of Laboratory Animal Resources, Seoul National University (SNU-140328-2). Unless otherwise stated, we obtained all chemicals from Sigma–Aldrich Corp. (St. Louis, MO, United States).

### *In vitro* production of fertilized embryos

Ovaries of prepubertal gilts were obtained from a local slaughterhouse and transferred to the laboratory within warmed saline. Cumulus-oocyte complexes (COCs) were collected by aspirating 3- to 7-mm follicles from the prepubertal gilts with a 10-ml syringe and an 18-gauge needle. COCs with compact multiple layers of cumulus cells and fine cytoplasm were collected from aspirated porcine follicular fluid (pFF) and allowed to mature for 44 h at 39°C in tissue culture medium 199 (TCM 199) (Gibco, Grand Island, NY, United States) supplemented with 10% pFF, L-cysteine (0.1 mg/ml), sodium pyruvate (44 ng/ml), epidermal growth factor (10 ng/ml), insulin (1 mg/ml), and kanamycin (75 μg/ml). The COCs were matured for the first 22 h with 10 IU/ml gonadotropin hormones, pregnant mare serum gonadotropin (PMSG) (Lee Biosolutions, Maryland Heights, MO, United States), and human chorionic gonadotropin (hCG); the gonadotropins were excluded from the medium for the last 22 h. After maturation, cumulus cells were removed from the oocytes with hyaluronidase. Sperm cells were washed twice with Dulbecco’s phosphate buffered saline (DPBS) supplemented with 0.1% bovine serum albumin (BSA) at 1,400 rpm for 3 min. Washed sperm (4 × 10^4^/ml in the final concentration) were co-incubated with the matured oocytes in 500 μl of modified tris-buffered medium (mTBM) for 4 h (Abeydeera and Day, 1997). mTBM consisted of 113.1 mM sodium chloride, 3 mM potassium chloride, 7.5 mM calcium chloride, 20 mM Trizma® base, 11 mM glucose, 5 mM pyruvate, 1 mM caffeine, and 0.8% BSA. Following this process, the eggs were incubated in 5% CO_2_ and 5% O_2_ at 39 °C in 20 μl of porcine zygote medium 3 (PZM3) (Yoshioka et al., 2002).

### Culture of cells from blastocyst seeding (including AP staining)

Hatched blastocysts were attached to feeder cells (mitomycin C-treated mouse embryonic fibroblasts). In total, 12 to 24 blastocysts were used in each seeding experiment. The basal medium was DMEM/F-12 supplemented with MEM Non-Essential Amino Acids, Glutamax, 2-mercaptoethanol, antibiotic-antimycotic, and 15% KnockOut™ Serum Replacement (all from Gibco, NY, United States). Basic FGF and human LIF were added to the medium (10 ng/ml each). AG1296 (PDGF receptor inhibitor, 10 μM), SB431542 (TGFβ inhibitor, 4 μM), and PD0325901 (MEK inhibitor, 1 μM) were used for the inhibitor treatment experiment. Samples were cultured for 14 days after blastocyst seeding and stained with alkaline phosphatase (AP).

### Immunocytochemistry of colonies

Cells were washed with DPBS supplemented and fixed with 4% paraformaldehyde (PFA) in DPBS at room temperature (RT) for 15 min. Fixed cells were permeabilized using 0.2% Tween-20 and 0.1% Triton X-100 in DPBS at RT for 15 min, followed by blocking with 10% donkey serum in DPBS at RT for 1 h. Samples were stained with anti-SOX2 (5 μg/ml) or anti-GATA6 (1 μg/ml) in DPBS containing 10% donkey serum at 4°C overnight. After washing three times in washing solution (DPBS with 0.2% Tween-20 for 10 min), cells were incubated with donkey anti-rabbit Alexa594 or donkey anti-goat Alexa488 (Invitrogen 1:5,000) in DPBS with 10% donkey serum at RT for 1 h and at 4 °C for 6 h. For double staining, samples were stained again with anti-SOX17 (1 μg/ml) or anti-NANOG (1 μg/ml). The procedures were the same as for the first staining. The antibodies used are listed in Table 1. A digital imaging system for microscopy (DS-L1, Nikon) was used to obtain fluorescence and bright-field images. We used ImageJ software to process the images.

**TABLE 1:** List of antibodies.

Primary antibodies			
Target	Host	Company	Catalog number
SOX SOX172	Goat	R&D systems	AF1924
SOX2	Rabbit	Millipore	AB5603
GATA6	Goat	R&D systems	AF1700
NANOG	Rabbit	Peprotech	500-P236
**Secondary antibodies**			
**Fluorescent dye**	**Target/host**	**Company**	**Catalog number**
Alexa594	Rabbit/donkey	Invitrogen	A-21207
Alexa488	Goat/donkey	Invitrogen	A-11055

### RNA extraction and quantitative PCR

Each cell population from the colonies was mechanically separated from the blastocyst seeding samples. RNA was extracted and cDNA was synthesized with a TaqMan™ Gene Expression Cells-to-CT™ Kit (Invitrogen, MA, United States). Quantitative PCR was conducted using Power SYBR™ Green PCR Master Mix (Applied Biosystems™, CA, United States). The levels of the transcripts were normalized to the expression level of the GAPDH gene. The list of primers used is described in Table 2.

**TABLE 2:** List of oligonucleotides for quantitative PCR.

Cell lineage	Gene	Forward sequence	Reverse sequence	Annealing temperature (°C)	Product size (base pairs)
Reference	*GAPDH*	TGCTCCTCCCCGTTCGAC	ATGCGGCCAAATCCGTTC	60	100
Epiblast	*OCT4A*	CTT​GGA​GAG​CCC​TGG​TTT​TAC​T	GCC​AGG​TCC​GAG​GAT​CAA​C	68	159
	*SOX2*	CGGCGGTGGCAACTCTAC	TCG​GGA​CCA​CAC​CAT​GAA​AG	64	100
	*NANOG*	CAT​CTG​CTG​AGA​CCC​TCG​AC	GGG​TCT​GCG​AGA​ACA​CAG​TT	60	195
	*HNF4A*	GCT​TCT​TTC​GGA​GGA​GTG​TG	TTG​ACC​TGC​GAG​TGC​TGA​T	60	183
	*KLF4*	GGA​CCA​CCT​TGC​CTT​ACA​CA	CTT​TCC​AGC​TGG​GTT​CCT​CC	60	146
	*MYC*	GAA​AAA​GAC​GTG​CTG​CGG​AA	CCA​GCC​AAG​GTT​GTG​AGG​TT	60	253
PrE	*PDGFRA*	GGT​CAC​CTG​TGC​CGT​CTT​TA	TTT​GAT​GGA​CGG​GAC​CTT​GG	60	115
	*PDGFA*	GCT​GTG​GAT​ACC​TCG​CCA​AT	CTT​CTC​TTC​CTC​CGA​ACG​GG	60	132
	*SOX17*	GCAAGATGCTGGGCAAGT	TTG​TAG​TTG​GGG​TGG​TCC​TG	60	112
	*GATA4*	GACCACCACCACCACGCT	AAT​CCC​CTC​TTT​CCG​CAT​T	60	121
	*GATA6*	CGG​CCT​CTA​CAG​CAA​GAT​GA	AGT​TGG​CAC​AGG​ACA​ATC​CA	60	98
TE	*CDX2*	CAGCGGCGGAACCTGTG	ACT​CGG​TAT​TTG​TCT​TTC​GTC​CTG	63	92
	*DAN2*	TGG​GAG​TGA​GGC​CCT​AAT​GA	GGA​CTA​CTT​AGG​TCG​GGA​GGT	60	111
	*GATA3*	GCGGGCTCTACCACAAAA	CGT​TGG​CAT​TTC​TTC​TCC​A	60	141
XEN	*SALL4*	CAG​GAG​TAC​CAG​AGC​CGA​AG	ACC​TCG​GGA​GAC​TTG​GAC​TT	60	107
	*SNAI1*	TTT​TCA​GCA​GCC​CTA​TGA​CC	CCA​GGA​GAG​AGT​CCC​AGA​TG	60	107
	*SPARC*	GGA​CCA​TCA​GTC​CTC​TGG​AA	AGT​TCT​GCG​TCT​CCC​AAA​GA	60	111
Mesoderm	*T*	GGG​CAA​GGG​ATG​GGA​ATA​AGG	ACC​GCT​GAG​GAT​GGA​CAA​AG	60	112
	*GSC*	GAA​GCC​CTG​GAG​AAC​CTC​TT	GCT​TTC​GAC​GAC​GTC​TTG​TT	60	200
	*GATA5*	GAAACCCGAGCCCAGCC	GGA​GTG​AAG​AGG​CAG​CGA​G	60	172
	*MIXL1*	AGA​TGT​GAA​CTG​CCT​GCC​C	ATT​CTG​GTG​TGT​GTC​TCC​CTG	60	232
Germ cell	*IFITM3*	TTC​GTG​GCT​TTC​GCC​TAC​TC	CCA​GTG​GTG​CAA​ACG​ATG​AT	60	161
	*DDX4*	GAA​CCC​AGT​TGG​GGC​ATT​CA	TTT​GAT​GGC​ATT​CCT​GGG​CA	64	211
	*PRDM1*	GTT​CAG​GCA​GAG​GCA​TCC​TT	GAG​TGT​GCT​GGG​TTC​ACG​TA	60	272
	*PTEN*	CCA​GTC​AGA​GGC​GCT​ATG​TG	TGG​CAG​ACC​ACA​AAC​TGA​GG	64	151

### RNA sequencing

RNA was purified from each type of cell (Clear-S™, Invirustech, Korea). Libraries were prepared using the SMART-Seq® v4 Ultra® Low Input RNA Kit for Sequencing (Takara Bio, CA, United States). An Illumina Novaseq 6000 (CA, United States) was used to produce read counts of samples. For the comparative study, previous reports were used (Table 3). A list of the tools used for the analysis is given in Table 4.

**TABLE 3:** List of RNA-seq data.

Access number of GEO	Name of sample	Run	Description	Sample name in this study
GSE189477	GSM5702418	Not published (private until publication)	Epiblast-like cells	Type A (A)
	GSM5702419	Not published (private until publication)	Epiblast-like cells	Type A (A1)
	GSM5702420	Not published (private until publication)	Epiblast-like cells	Type A (A2)
	GSM5702421	Not published (private until publication)	Primitive endoderm-like cells	Type B (B)
	GSM5702422	Not published (private until publication)	Primitive endoderm-like cells	Type B (B1)
	GSM5702423	Not published (private until publication)	Primitive endoderm-like cells	Type B (B2)
	GSM5702424	Not published (private until publication)	Trophectoderm-like cells	Type C (C)
	GSM5702425	Not published (private until publication)	Trophectoderm-like cells	Type C (C1)
	GSM5702426	Not published (private until publication)	Trophectoderm-like cells	Type C (C2)
	GSM5702427	Not published (private until publication)	Mesoderm-like cells	Type D (D)
	GSM5702428	Not published (private until publication)	Mesoderm-like cells	Type D (D1)
	GSM5702429	Not published (private until publication)	Mesoderm-like cells	Type D (D2)
GSE120031	GSM3391893	SRR7851658	Pig fetal fibroblasts	PEF
	GSM3391894	SRR7851659	Pig fetal fibroblasts	PEF
	GSM3391895	SRR7851660	Pig fetal fibroblasts	PEF
	GSM3391902	SRR7851667	Pig ESCs	IVF-ES
	GSM3391903	SRR7851668	Pig ESCs	IVF-ES
	GSM3391904	SRR7851669	Pig ESCs	IVF-ES
GSE112380	GSM3069020	SRR6904203	Pig embryo: Morula	Morula
	GSM3069022	SRR6904205	Pig embryo: Morula	Morula
	GSM3069025	SRR6904208	Pig embryo: Morula	Morula
	GSM3069058	SRR6904241	Pig embryo: EB ICM	EB_ICM
	GSM3069061	SRR6904244	Pig embryo: EB ICM	EB_ICM
	GSM3069065	SRR6904248	Pig embryo: EB ICM	EB_ICM
	GSM3069081	SRR6904264	Pig embryo: EB TE	EB_TE
	GSM3069092	SRR6904275	Pig embryo: EB TE	EB_TE
	GSM3069094	SRR6904277	Pig embryo: EB TE	EB_TE
	GSM3069096	SRR6904279	Pig embryo: LB EPI	LB_Epi
	GSM3069113	SRR6904296	Pig embryo: LB EPI	LB_Epi
	GSM3069118	SRR6904301	Pig embryo: LB EPI	LB_Epi
	GSM3069131	SRR6904314	Pig embryo: HYPO	LB_PrE
	GSM3069134	SRR6904317	Pig embryo: HYPO	LB_PrE
	GSM3069142	SRR6904325	Pig embryo: HYPO	LB_PrE
GSE66507	GSM1624222	SRR1825955	Human embryo: TE	TE
	GSM1624225	SRR1825958	Human embryo: TE	TE
	GSM1624228	SRR1825961	Human embryo: EPI	Epiblast
	GSM1624229	SRR1825962	Human embryo: TE	TE
	GSM1624232	SRR1825965	Human embryo: PE	PrE
	GSM1868810	SRR2240580	Human embryo: PE	PrE
	GSM1868810	SRR2240581	Human embryo: PE	PrE
	GSM1868823	SRR2240644	Human embryo: EPI	Epiblast
	GSM1868823	SRR2240645	Human embryo: EPI	Epiblast

**TABLE 4:** Tools and parameters.

Tools_1	Name of tool	Version	Parameter	Reference
Preprocessing				
Low-quality reads and adapter sequences were filtered according to the following parameters	cutadapt	2.8	quality-cutoff (20) and minimum-length (50)	Martin, (2011)
Alignment				
Filtered reads were mapped to the reference genome related to the species. The parameters have been set based on ENCODE standard options.	STAR	2.7.1a	outFilterType (BySJout), outFilterMultimapNmax (20), alignSJoverhangMin (8), alignSJDBoverhangMin (1), outFilterMismatchNmax (999), outFilterMismatchNoverLmax (0.04), alignIntronMin (20), alignIntronMax (1000000), and alignMatesGapMax (1000000)	Dobin et al. (2013)
Abundance estimation				
The expression levels of genes and transcripts were calculated using the read mapping information obtained from the aligner in the following manner	RSEM	1.3.1	—	Li and Dewey, (2011)
	featureCounts	2.0.0	—	Liao et al. (2014)
	HTSeq-count	0.11.2	minaqual (0) and mode (intersection-nonempty)	Anders et al. (2015)
	Cufflinks	2.2.1	multiread-correct and frag-bias-correct	Trapnell et al. (2010)
Tools_2		Name of the tool	Version	Ref
Differentially expressed gene (DEG) analysis				
Using genes and transcript expression information, genes were predicted with statistically significant differences in expression between samples or groups		TCC	1.26.0	Sun et al. (2013)
		edgeR	3.28.1	Robinson et al. (2010)
		DESeq	1.38.0	Anders and Huber, (2010)
		DESeq2	1.26.0	Love et al. (2014)
		EnhancedVolcano	-	Kevin et al. (2018)
Functional study				
The biological function of DEGs is being investigated to identify biological clues for research using differences in the expression levels		goseq	1.38.0	Young et al. (2010)
		GOplot	1.0.2	Walter et al. (2015)
Data quality control (QC)				
Data QC was performed to verify sequencing and alignment data, etc.		FastQC	0.11.9	Andrews, (2010)
		PCAtools	—	Kevin Blighe, (2019)
		Gviz	1.30.0	Hahne and Ivanek, (2016)

### Statistical analysis

Statistical analysis of the data was performed using GraphPad Prism Software (version 5.01; San Diego, CA, United States). Significant differences among experimental groups were determined by one-way analysis of variance (ANOVA) followed by Tukey’s multiple comparison test, while unpaired t-tests were used for the binomial data. A *p*-value < 0.05 was considered significant. Data are presented as the mean± the standard error.

## Results

### Morphology and protein localization in each type of cell

**FIGURE 1:**
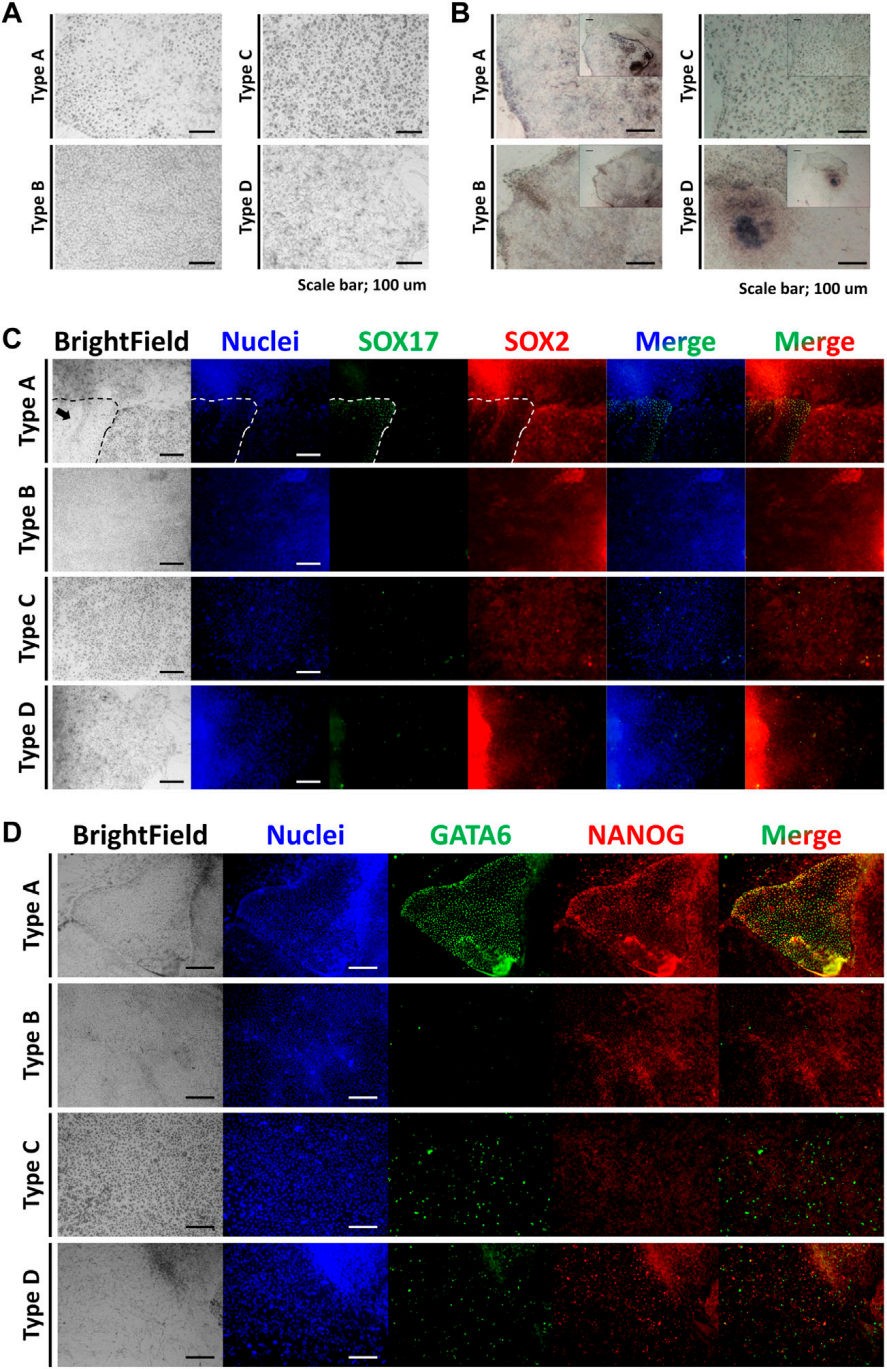
Morphologies, alkaline phosphatase staining, and immunocytochemistry of the four types of cells. **(A)** Morphologies of the four cell types. **(B)** Alkaline phosphatase staining of the four cell types. **(C)** Immunocytochemistry staining images of the four cell types for SOX17 and SOX2 (the arrow indicates the area of type A cells). **(D)** Immunocytochemistry staining images of the four cell types for GATA6 and NANOG. All scale bars are 100 μm.

The morphological features of each cell population are shown in Figure 1A. Types A, B, and C cultured as single layers, but type D formed multiple layers. Lipid droplets (LDs) were determined by microscopy with normal light, following a previous study (Niu et al., 2015). Cell types A and C contained LDs, while B and D did not show LDs. The density order of the cells was type D, B, A, and C. In the AP treatment experiment, type A cells were stained, but cell types B and C were not AP-positive. Within type D, the central part of the colony (high cellular density) showed an AP-positive signal (Figure 1B). Because the type D cells grew in multiple layers, the AP staining always showed the same pattern as shown in Figure 1B. SOX17 protein was detected only in the nuclei of type A cells. SOX2 was localized in the nuclei of type A, B, and D cells, but this protein was localized in the cytoplasm of type C cells (Figure 1C; Supplementary Figure S1). Similar to SOX17, GATA6 was detected in the nuclei of type A cells. Type B cells were negative for GATA6. Cell types C and D showed a positive signal for GATA6, but it was not negative in type B. NANOG was observed in the nuclei of type A and B cells, while it was observed in the cytoplasm of type C cells (Figure 1D). All of the results from this section are summarized in Figure 3A, and representative images are shown in Figure 3B.

### Relative expression level of lineage-specific marker genes in each type of cells

**FIGURE 2:**
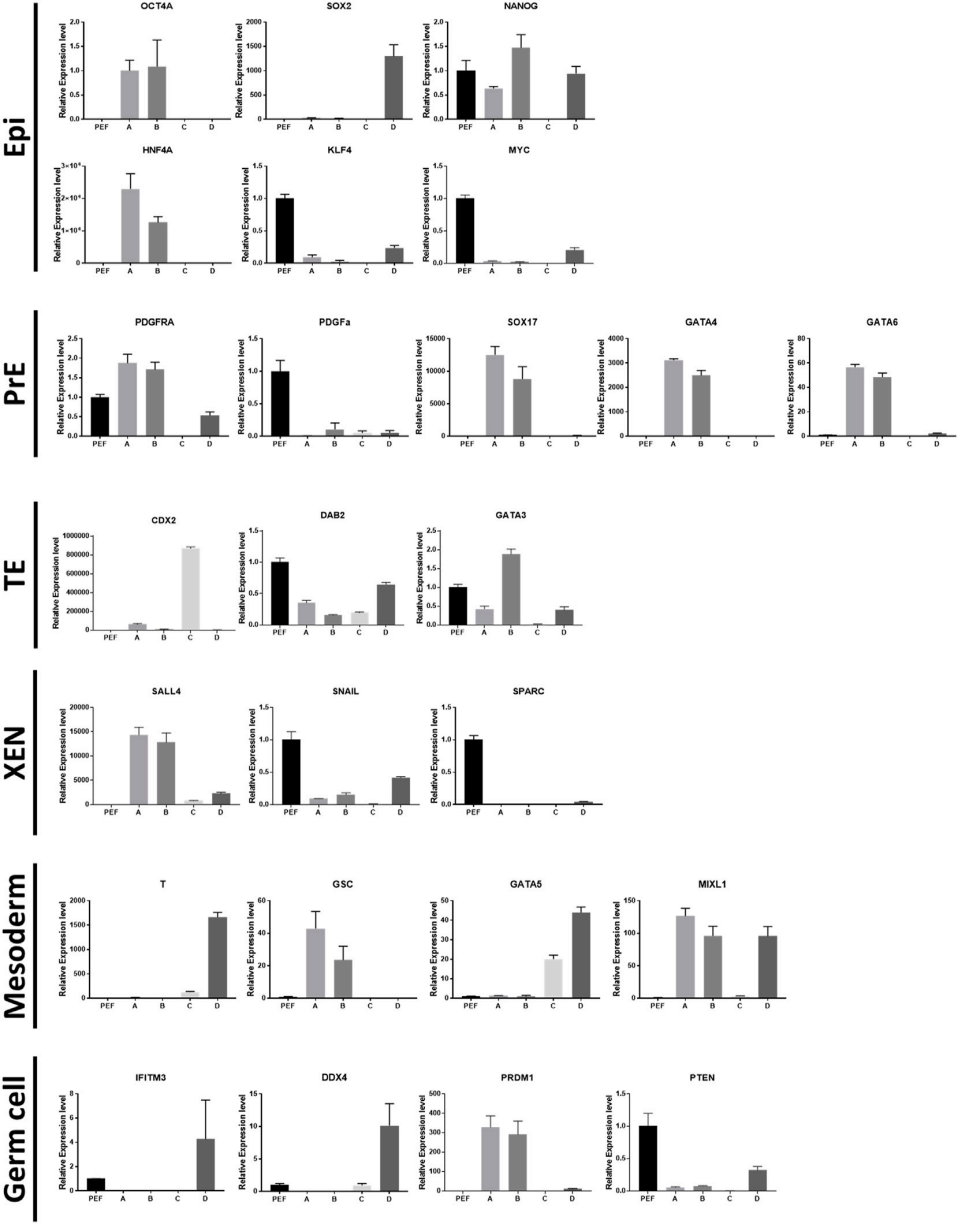
Relative expression levels of lineage marker genes. Epiblast: OCT4A, SOX2, NANOG, HNF4A, KLF4, MYC. Primitive endoderm: PDGFRA, PDGFA, SOX17, GATA4, GATA6. Trophectoderm: CDX2, DAB2, GATA3. Extraembryonic endoderm cells: SALL4, SNAI1, SPARC. Mesoderm: T, GSC, GATA5, MIXL1. Germ cells: IFITM3, DDX4, PRDM1, PTEM.

**FIGURE 3:**
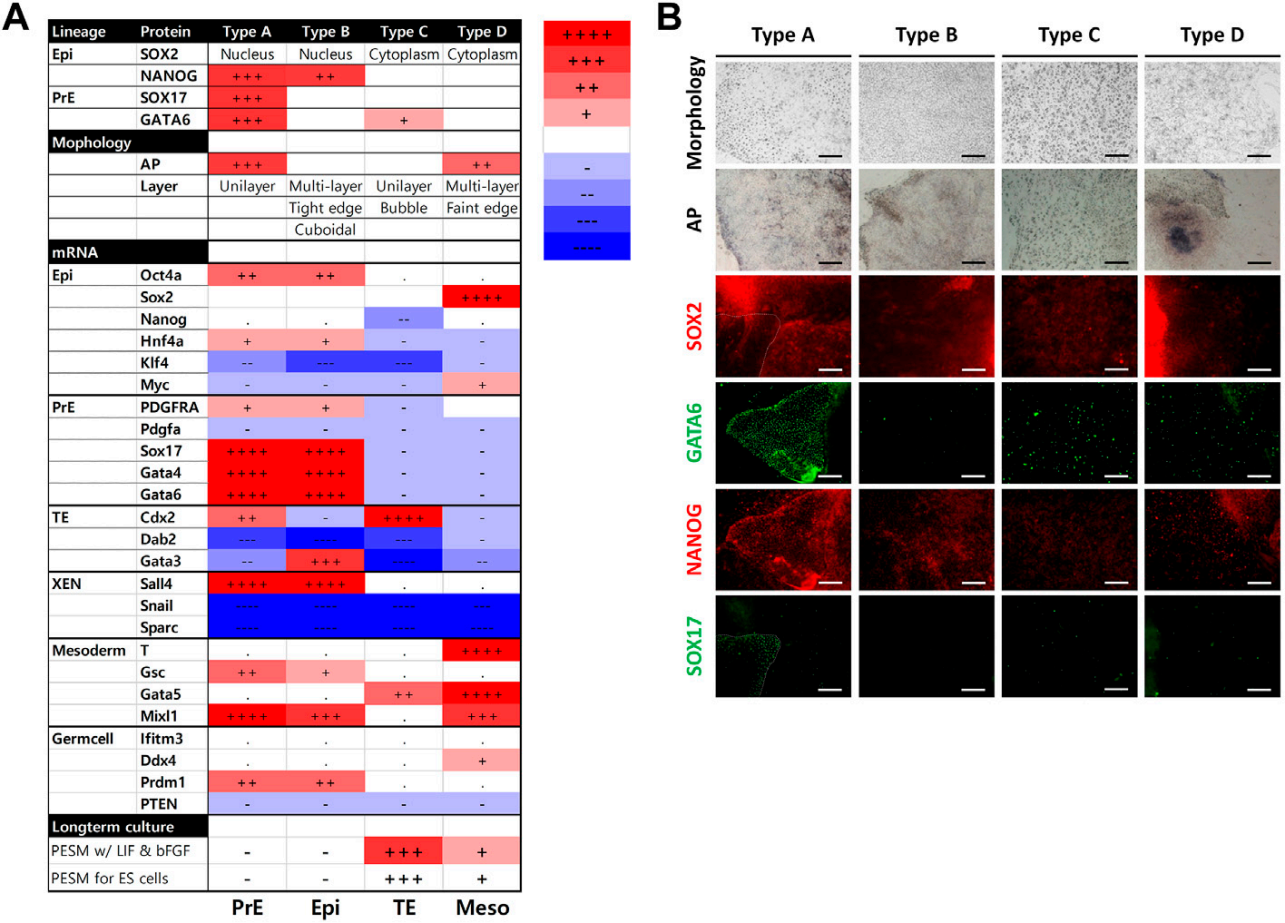
Summary of the results from the four types of cells. **(A)** Summary of characteristics for the cell types. mRNA levels were determined using porcine embryonic fibroblasts as a control. The number of + or − represents the level of statistical significance. (+ or −, *p* < 0.05; ++ or −, *p* < 0.01; +++ or −, *p* < 0.001; ++++ or−, *p* < 0.0001) **(B)** Summary of bright-field and immunocytochemistry images. Images were collected from Figure 1.

Relative expression levels of marker genes were normalized with porcine embryonic fibroblasts (PEFs) as a control (Figure 2). Among the epiblast markers, Oct4a and Hnf4a showed high levels of expression in cell types A and B. The expression of Sox2 was restricted in type D cells. Nanog expression in type C cells showed significantly lower expression than that in PEFs. The expression levels of Klf4 and Myc were lower in the four types of cells than in somatic cells. PDGFRA expression was high in type A and B cells, but PDGFα expression was lower in the four cell types than in PEFs. Other PrE-specific genes, Sox17, Gata4, and Gata6, were highly expressed in cell types A and B. Among TE-specific markers, Cdx2 showed specific expression in type C cells. Dab2 expression in the four cell types was lower than that in PEFs, while Gata3 expression was only high in type B cells. Among XEN cell markers, Snail and Sparc expression in the four types of cells was lower than that in PEFs. Sall4 was highly expressed in cell types A and B. Mesoderm marker T showed exclusive expression in type D cells. Type D cells also showed the highest expression of Gata5. Additionally, the expression of Gata5 in type C cells was lower than that in type D cells but higher than that in cell types A and B. Gsc expression was high in cell types A and B, and the expression of Mixl1 was significantly high in cell types A, B, and D. In the case of germ cell markers, the expression levels of Prdm1 were significantly high in cell types A and B. However, Ddx4 expression was only higher in cell type D than in the other cell types. Moreover, Pten expression in the four cell types was lower than that in PEFs. All of the results from this section are summarized in Figure 3A.

### Treatment of colonies with signaling inhibitors

**FIGURE 4:**
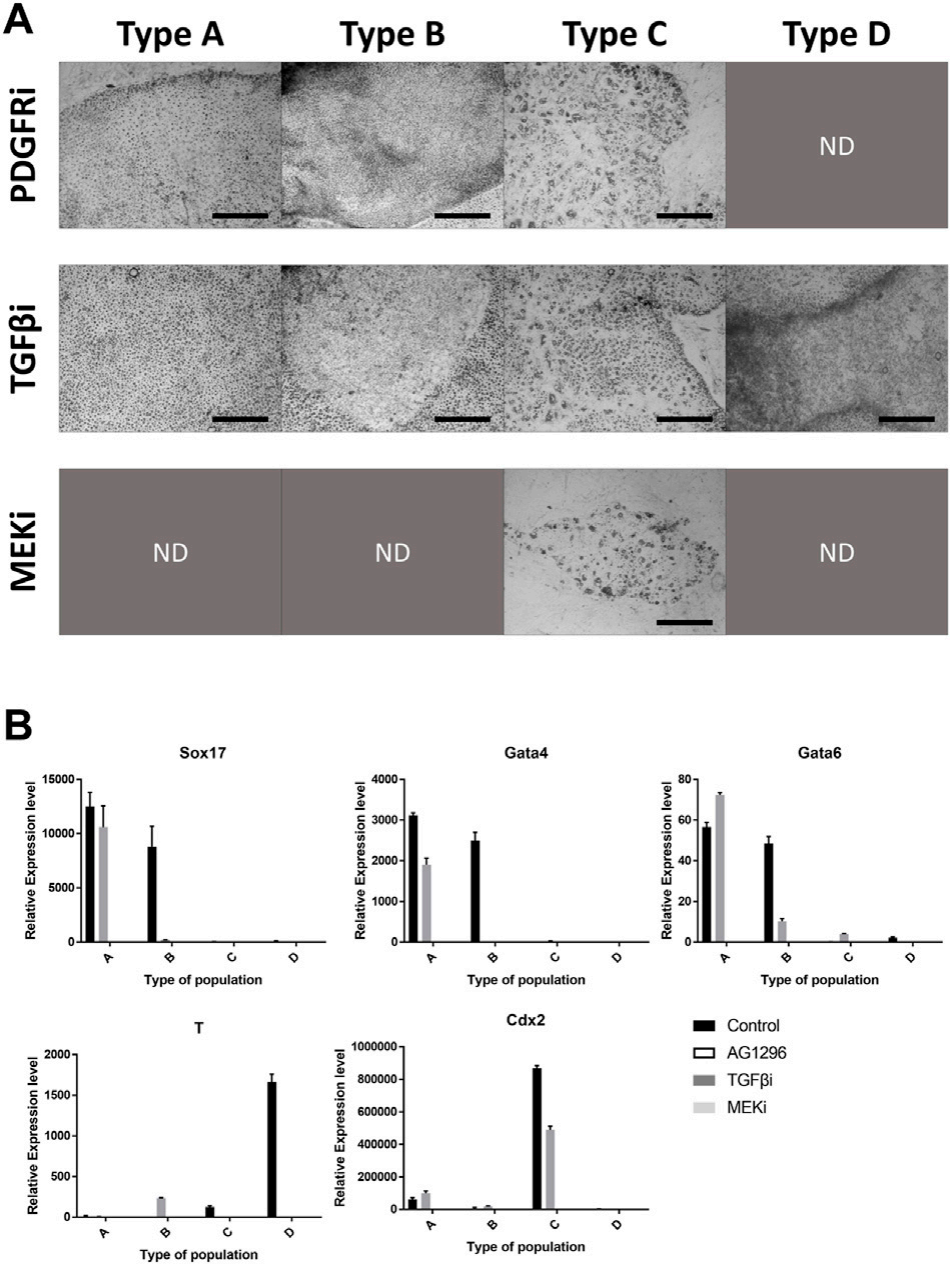
Effects of signaling inhibitors on the four types of cells. **(A)** Morphology of each cell type with inhibitor-treated cell types. Images were collected from Figure 5. **(B)** Expression patterns of marker genes in cell types with inhibitor treatment. All scale bars are 100 μm. Different letters show significant differences among samples. (PDGFRi, platelet-derived growth factor receptor inhibitor; TGFβi, transforming growth factor β inhibitor; MEKi, mitogen-activated protein kinase inhibitor; ND, not detected).

**FIGURE 5:**
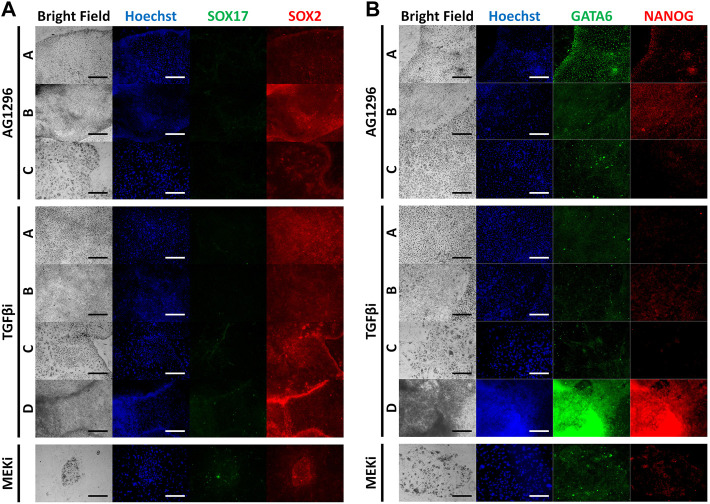
Immunocytochemistry images of inhibitor-treated cell types for marker proteins. **(A)** Immunocytochemistry staining of SOX17 and SOX2 with inhibitor-treated cell types. **(B)** Immunocytochemistry staining of GATA6 and NANOG with inhibitor-treated cell types. (TGFβi, transforming growth factor β inhibitor; MEKi, mitogen-activated protein kinase inhibitor).

The inhibitors we used were powerful, therefore some cell types were not detected after the culture period (Figure 4A). The four types were observed following TGFβ inhibitor treatment. However, AG1296 suppressed the development of cell type D, and only type C cells survived MEK inhibitor treatment. We selected five marker genes (Sox17, Gata4, Gata6, T, and Cdx2) from the above experiment (Figure 4B). In type A cells, PrE markers were greatly reduced by a TGFβ inhibitor, and these markers were decreased in type B cells by AG1296. The TE marker Cdx2 in type C cells was downregulated by all three inhibitors; in particular, the MEK inhibitor strongly suppressed Cdx2. The mesoderm marker T was repressed in type D cells by a MEK inhibitor. Next, the cells were immunostained with the antibodies listed in Figure 5. No difference was detected in the pattern of SOX2 following inhibitor treatment, but the SOX17 signal was not observed under all conditions. In the case of GATA6 and NANOG, AG1296 and MEK inhibitors did not affect the pattern of protein expression. Under TGFβ inhibitor treatment, the nucleus-specific positive signals of GATA6 and NANOG in type A cells disappeared, and NANOG was translocated to the cytoplasm in type B cells. Additionally, type C cells were not positive for either protein.

### RNA sequencing of the four cell types

**FIGURE 6:**
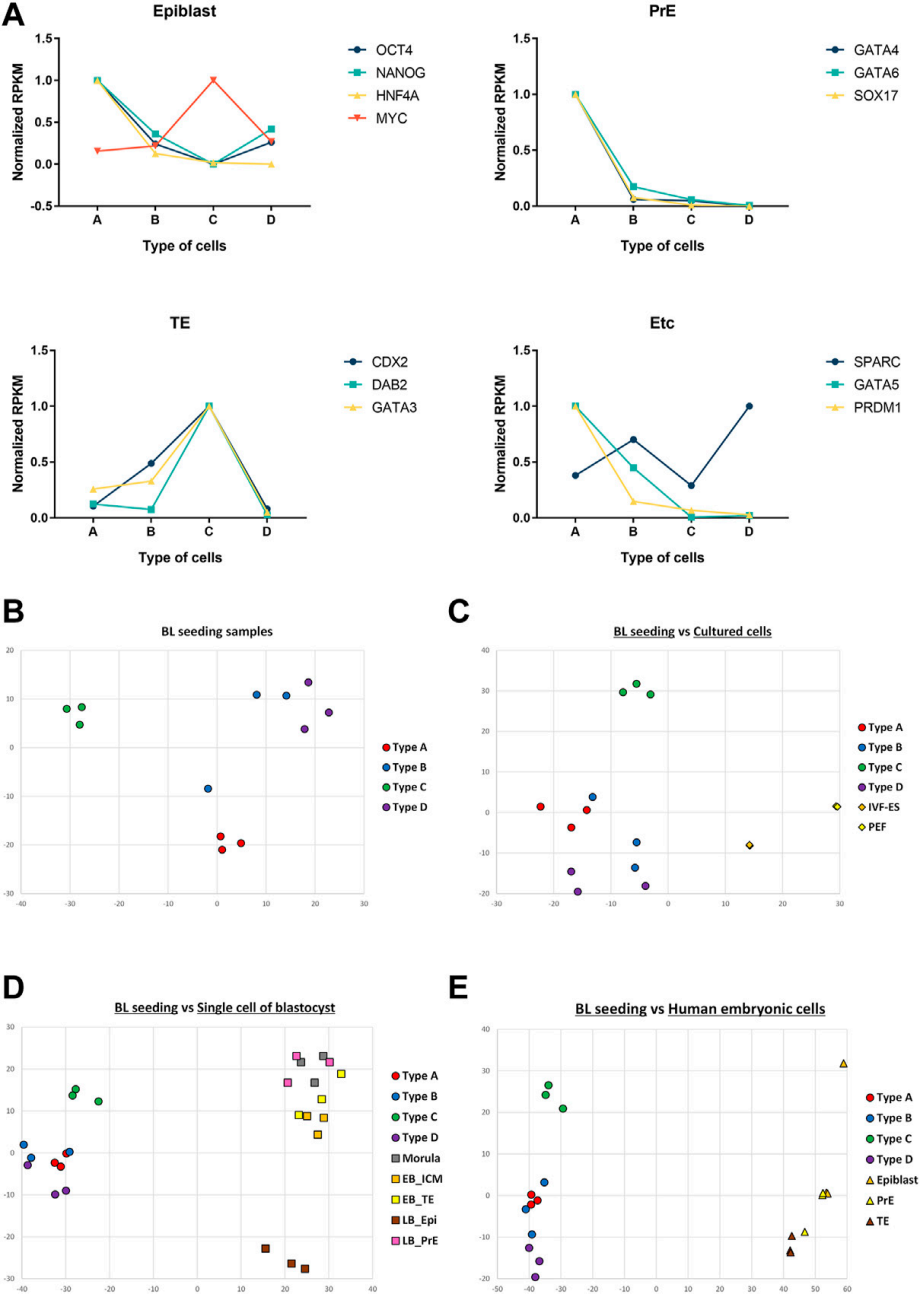
RNA-seq data of the four types of cells and comparison of RNA-seq results. **(A)** Normalized RPKM values of marker genes. Epiblast: OCT4, NANOG, HNF4A, MYC. Primitive endoderm: GATA4, GATA6, SOX17. Trophectoderm: CDX2, DAB2, GATA3. XEN; SPARC. Mesoderm: GATA5, Germ cell: PRDM1. Multidimensional scaling of samples. **(B)** Multidimensional scaling of 12 samples from the four cell types. **(C)** Comparison of 12 samples with cultured porcine cells. **(D)** Comparison of 12 samples with single-cell RNA-seq results from porcine embryos. **(E)** Comparison of 12 samples with single-cell RNA-seq results from human embryos. (BL, blastocysts; IVF-ES, embryonic stem cells established from *in vitro* fertilized embryos; PEF, porcine embryonic fibroblasts; EB, early blastocysts; LB, late blastocysts; ICM, inner cell mass; TE, trophectoderm; Epi, epiblasts; PrE, primitive endoderm).

A summary of the RNA sequencing is given in Supplementary Table S1. Raw data were aligned with reference genome information of pigs (Supplementary Table S2). Mapped reads were categorized by the value of reads per kilobase per million (RPKM) (Supplementary Table S3). We found up- or down-regulated genes in each cell type (Table 5). Only triplicates were used for each type, so no significant differences were detected in the lineage marker results. However, to show any tendencies of the marker genes, relative RPKMs were visualized (Figure 6A). Of the epiblast markers, Oct4, Nanog, and Hnf4a showed peaks in cell type A. All the PrE markers had the highest value in cell type A. The TE markers Cdx2, Dab2, and Gata3 showed peaks in cell type C. Correlations among the 12 samples were analyzed by multidimensional scaling (Figure 6B). Samples were gathered according to their types. This result means that each type showed a distinct pattern of RNA expression. We also compared 12 samples with RNA sequencing data of previous studies (Figure 6C). In the comparison with porcine embryonic stem cells and somatic cells, the 12 samples showed a closer correlation with embryonic stem cells than somatic cells. Next, the 12 samples were compared with single-cell RNA-seq samples of porcine and human embryos (Figures 6D,E, respectively). In this analysis, no correlation was found between our samples and the single-cell RNA-seq data.

**TABLE 5:** List of type-specific up- or down-regulated genes from the four cell types.

Sample	Upregulated genes	Downregulated genes
Type A	KRT8, APOE, RBP4, TF, FETUB, GPC3, CLDN6, P3H1, MDH1, APOA1, GSN, ISYNA1, CKB	
Type B	CXCL14, COX3, S100A6, CRABP1, APOA1, ENSSSCG00000032599, ENSSSCG00000034846, ENSSSCG00000041596, ENSSSCG00000041875	
Type C	LGMN, S100A6, TACSTD2, ANXA2, B2M, CLDN4, CST6, PTGS2, MEST, ENSSSCG00000017061, GRN, COX1, CD9, MT1A, ENSSSCG00000024911, PLBD1, CSTB, TIMP3, PLET1, ENSSSCG00000035724, ENSSSCG00000037567, KRT7, HSPB1, CTSD, ENSSSCG00000048235	TMSB10, MDK, PRDX2, ENSSSCG00000014540, UBB, ENO1, H3-3A, ENSSSCG00000032003, IGFBP2
Type D	CKB, CRABP1, HMGN2, S100A11, TMSB10, H2AFZ, ENSSSCG00000009327, ENSSSCG00000012119, MDK, PRDX2, ENSSSCG00000017202, UBB, COX2, ATP8, COX3, ENO1, H3-3A, STMN1, ENSSSCG00000032599, ENSSSCG00000034846, PTMA, ENSSSCG00000039506, ID3	KRT8, KRT18

## Discussion

Unlike previous studies, we tried to produce multiple types of embryonic cells with minimum signaling chemicals. We used both hLIF and bFGF together with the recipe described in our previous report (Choi et al., 2019). We induced four types of cell populations using a single protocol, and their characteristics were analyzed.

From the patterns of marker proteins and profiles of gene expression, we concluded that these four cell types could represent four embryonic lineages of early blastocysts (type A, PrE; type B, epiblast; type C, TE; type D, mesoderm). First, we could easily classify the types based on their morphology. Well-known protein markers of pluripotent cells, SOX2, GATA6, NANOG, and SOX17, showed their availability as standards to distinguish the four types. Additionally, among candidate genes, some genes demonstrated that their expression levels could be used as type-specific markers of gene expression. The nuclei of type A cells were highly positive for the PrE-specific markers SOX17 and GATA6. Also, similar to PrE tissue in embryos, type A cells only grew in a single layer. Type B cells shared many marker patterns with type A cells, including NANOG protein and mRNA expression. One of the major differences between cell types A and B was morphology. Type B cells could grow in multiple layers with cuboidal shapes. In the early stages of embryonic development, only epiblast cells can have many layers between two monolayer cell types, TE and PrE. Additionally, early epiblast cells have an apolar cell shape similar to that of the type B cells (Sheng, 2015). SOX2 is known as a marker of pluripotent cells such as ICM cells and epiblasts (Avilion et al., 2003). However, SOX2 has also been detected in the cytosol of TE and cancer cells (Keramari et al., 2010; Artus et al., 2011). Our results corresponded with those reports. Type C cells showed similar characteristic to TE cells. This type only expanded in the monolayer, and when the density of cells became too high, it detached from the feeder cells to form a bubble-like structure. The strong mesoderm marker T was only expressed in type D cells among the four cell types, and these cells were AP-positive (Herrmann et al., 1990). Therefore, type D cells were assumed to be a mesoderm lineage.

AG1296, an inhibitor of the PDGF receptor, repressed the induction of type D cells during the culture period. Type A cells maintained GATA6-positivity but lost SOX17 intensity. TGFβ inhibitors and MEK inhibitors are well-known cytokines that suppress the epiblast lineage in embryos and embryonic stem cells (Vrbsky et al., 2015). The four cell types we cultured can be produced with TGFβ inhibitor. However, type A cells lost the expression of both SOX17 and GATA6. In our results, the MEK inhibitor was a much stronger regulator than TGFβ in porcine embryos. Only type C-like cells could be obtained, while the expression level of Cdx2, a TE marker, was significantly reduced. Therefore, these cells could be suggested as type C, whereas it is hard to conclude them as type C cells. Treatment of AG1296 also resulted in changes in marker gene expression.

Based on the RNA-seq results, comparitive analyses were conducted. Up- or down-regulated genes were selected for each type of cell. Epiblast and TE markers showed peaks in epiblast-like type A and TE-like type D cella, respectively. Together with the qPCR and ICC data, type A could be inferred to be an epiblast lineage. Additionally, by the same token, type D cells might represent the TE lineage. However, the RPKM of PrE markers was highest for type A cells. In the case of PrE, the RNA-seq data were not consistent with the data above. The correlation of samples was examined, and a two-dimensional plot was produced. The same types of samples were clustered on the plot, but one of the type B samples was located near the type A samples. Mesoderm-like type D samples localized closer to type B than type A samples. During embryonic development, mesoderm cells originate from epiblasts, and PrE cells promote the induction of mesoderm formation. The developmental signal from PrE seemed to make mesoderm cells similar to PrE. The locations of TE-like type C samples were separate from the other samples. In the metadata analysis, we found that our samples were more like embryonic stem cells than somatic cells. However, because of the differences of data sources, single-cell RNA-seq data could not be used to characterize the four cell types in our study.

In conclusion, we have proposed a novel method for the induction of PrE, epiblasts, TE, and mesoderm-like cells from blastocysts using a single method. The limitation of suppressor molecules led to the survival of embryonic lineages during attachment culture. With additional inhibitors, some cell types were not observed, and the obtained cell types had transitions in marker proteins and mRNAs. Each cell type showed a unique response to signaling inhibitors. In our RNA-seq results, we found cell type-specific genes. Additionally, the similarity of the cell types was examined. As a further study, we are preparing type-specific culture conditions to maintain the characteristics of each cell type. Functional experiments such as differentiation of each cell type will be possible due to the extension of the culture period. This study will broaden the understanding of lineage specification in early embryos. In particular, we might develop a method to isolate cell populations in their natural state.

## Data Availability

The datasets presented in this study can be found in online repositories. The name of the repository and accession number can be found below: NCBI Gene Expression Omnibus; accession number GSE189477.
