# Supplementary figures and images for: Characterization of multitype colonies originating from porcine blastocysts produced in vitro
Source: Front Cell Dev Biol. 2022 Sep 12;10:918222. doi: 10.3389/fcell.2022.918222 (PMC9510650; doi:10.3389/fcell.2022.918222)

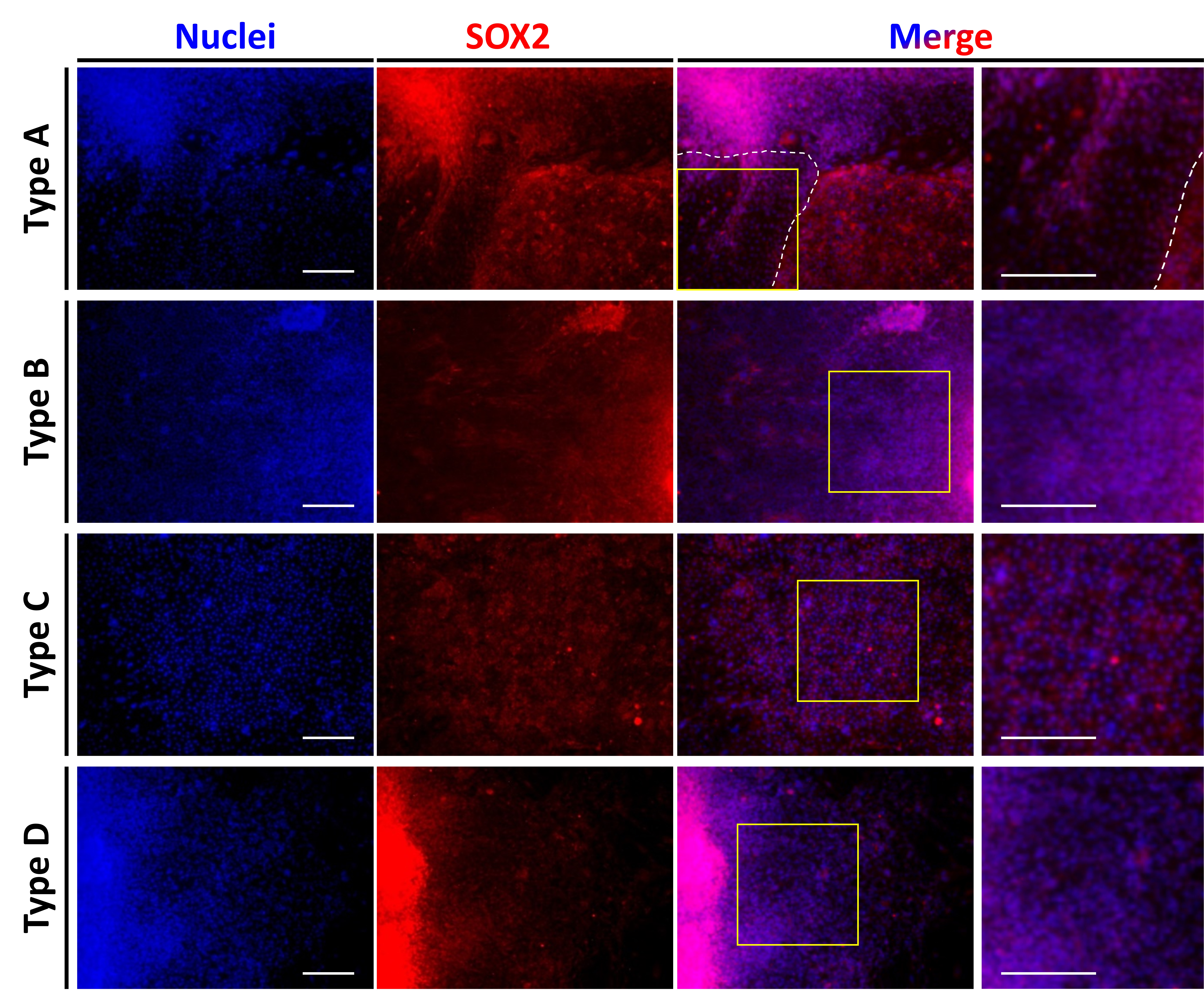

Supplement: Supplementary file 1 [file Image1.JPEG]
