# Supplementary material for: Characterization of multitype colonies originating from porcine blastocysts produced in vitro
Source: Front Cell Dev Biol. 2022 Sep 12;10:918222. doi: 10.3389/fcell.2022.918222 (PMC9510650; doi:10.3389/fcell.2022.918222)
Supplement: Supplementary file 2 [file DataSheet1.docx]

**Characterization of multi-type colonies originated from porcine in vitro produced blastocysts**

Jong-Nam Oh^1^, Jinsol Jeong^1^, Mingyun Lee^1^, Gyung Cheol Choe^1^, Dong-Kyung Lee^1^, Kwang-Hwan Choi^1^, Seung-Hun Kim^1^ and Chang-Kyu Lee^1, 2^

1 Department of Agricultural Biotechnology, Animal Biotechnology Major, and Research Institute of Agriculture and Life Sciences, Seoul National University, Seoul 08826, Korea

2 Designed Animal and Transplantation Research Institute (DATRI), Institute of Green Bio Science and Technology, Seoul National University, Pyeongchang 25354, Korea

**Figure s1. Immunocytochemistry of the four types of cells.**

The images which are indicated by “Nuclei” and “SOX2” are collected from Figure 1C. The rightest panels are magnified images from merged images. All scale bars are 100 μm.

**Table s1. Summary of RNA sequencing results**

Total Reads; Total number of produced reads, Total Bases; Total number of produced nucleotides, Total Bases(Gb); The total bases expressed by giga unit, GC Count; Number of both guanine(G) and cytosine(C) of the total bases, GC Rate; GC ratio of the total bases, N ZeroReads; Number of reads not including N(any base) nucleotide, N ZeroReadsRate; Proportion of the N ZeroReads of the total reads, N5 LessReads; Number of reads including less than 5 times of N(any base); nucleotide in each read, N5 LessReadsRate; Proportion of the N5 LessReads of the total reads, N Count; Total number of N(any base) nucleotides, N Rate; Proportion of the N Count of total bases, Q30 MoreBases; Number of nucleotides greater than Phred quality score 30 (nucleotide accuracy: 99.9%) of the total bases, Q30 MoreBasesRate; Proportion of the Q30 MoreBases of the total bases, Q20 MoreBases; Number of nucleotides greater than Phred quality score 20 (necleotide accuracy: 99%) of the total bases, Q20 MoreBasesRate; Proportion of the Q20 MoreBases of the total bases

| Sample  ID | TBI_ID | Total  Reads | Total  Bases | Total  Bases | GC_Count | GC_Rate | N_Zero  Reads | N_Zero  Reads Rate |
| --- | --- | --- | --- | --- | --- | --- | --- | --- |
| sample_A | TN2109R1732 | 38656488 | 5.84E+09 | 5.84 Gb | 2.78E+09 | 0.4768 | 38633058 | 0.9994 |
| sample_A1 | TN2109R1733 | 51516386 | 7.78E+09 | 7.78 Gb | 4.06E+09 | 0.5224 | 51493666 | 0.9996 |
| sample_A2 | TN2109R1734 | 48482874 | 7.32E+09 | 7.32 Gb | 3.82E+09 | 0.522 | 48460814 | 0.9995 |
| sample_B | TN2109R1735 | 43624090 | 6.59E+09 | 6.59 Gb | 3.47E+09 | 0.5262 | 43593992 | 0.9993 |
| sample_B1 | TN2109R1736 | 37813938 | 5.71E+09 | 5.71 Gb | 2.95E+09 | 0.5161 | 37786498 | 0.9993 |
| sample_B2 | TN2109R1737 | 43592668 | 6.58E+09 | 6.58 Gb | 3.13E+09 | 0.4761 | 43565072 | 0.9994 |
| sample_C | TN2109R1738 | 39024038 | 5.89E+09 | 5.89 Gb | 2.84E+09 | 0.4814 | 39002844 | 0.9995 |
| sample_C1 | TN2110R0393 | 66775832 | 1.01E+10 | 10.08 Gb | 4.94E+09 | 0.49 | 66724420 | 0.9992 |
| sample_C2 | TN2109R1740 | 48714776 | 7.36E+09 | 7.36 Gb | 3.55E+09 | 0.4831 | 48679256 | 0.9993 |
| sample_D | TN2109R1741 | 47682832 | 7.2E+09 | 7.20 Gb | 3.59E+09 | 0.4982 | 47647576 | 0.9993 |
| sample_D1 | TN2109R1742 | 42113266 | 6.36E+09 | 6.36 Gb | 3.27E+09 | 0.5136 | 42095328 | 0.9996 |
| sample_D2 | TN2109R1743 | 43945760 | 6.64E+09 | 6.64 Gb | 3.22E+09 | 0.4853 | 43922406 | 0.9995 |

| Sample  ID | N5_Less  Reads | N5_Less ReadsRate | N_Count | N_Rate | Q30_More Bases | Q30_More Bases Rate | Q20_More Bases | Q20_More Bases Rate |
| --- | --- | --- | --- | --- | --- | --- | --- | --- |
| sample_A | 38656428 | 1 | 14412 | 0 | 5.22E+09 | 0.8936 | 5.52E+09 | 0.9454 |
| sample_A1 | 51516306 | 1 | 14290 | 0 | 7.3E+09 | 0.9381 | 7.6E+09 | 0.9764 |
| sample_A2 | 48482808 | 1 | 13597 | 0 | 6.88E+09 | 0.9399 | 7.16E+09 | 0.9782 |
| sample_B | 43623950 | 1 | 19806 | 0 | 6.04E+09 | 0.9177 | 6.35E+09 | 0.9642 |
| sample_B1 | 37813802 | 1 | 18472 | 0 | 5.36E+09 | 0.9379 | 5.58E+09 | 0.9769 |
| sample_B2 | 43592540 | 1 | 17752 | 0 | 6.1E+09 | 0.9272 | 6.38E+09 | 0.9696 |
| sample_C | 39023972 | 1 | 12844 | 0 | 4.86E+09 | 0.8241 | 5.22E+09 | 0.8864 |
| sample_C1 | 66772906 | 1 | 119040 | 0 | 9.12E+09 | 0.9045 | 9.6E+09 | 0.9523 |
| sample_C2 | 48714612 | 1 | 23347 | 0 | 6.73E+09 | 0.9149 | 7.05E+09 | 0.9582 |
| sample_D | 47682654 | 1 | 23793 | 0 | 6.7E+09 | 0.9311 | 7E+09 | 0.9729 |
| sample_D1 | 42113208 | 1 | 11099 | 0 | 5.97E+09 | 0.9388 | 6.22E+09 | 0.9777 |
| sample_D2 | 43945670 | 1 | 14730 | 0 | 6.13E+09 | 0.9241 | 6.41E+09 | 0.9667 |

**Table s2. Alignment statistics of samples from four types**

Raw: Number of raw reads (Raw/Raw*100), Clean: Number of clean(preprocessed) reads (Clean/Raw*100), Mapped: Number of mapped reads (Mapped/Raw*100), Unique: Number of uniquely mapped reads (Unique/Raw*100), Spliced: Number of spliced reads (Spliced/Raw*100), InsertSizeAvg: Average of insert size, InsertSizeSD: Standard deviation of insert size

| Sample | ReadsRaw  Count | ReadsClean  Count | ReadsClean  Rate | Mapped  Reads | Mapped  Rate | Uniquely  MappedReads | Uniquely  MappedRate | Spliced  Reads | Spliced  Rate | InsertSize  Avg | Insert  SizeSD |
| --- | --- | --- | --- | --- | --- | --- | --- | --- | --- | --- | --- |
| A | 38656488 | 37667824 | 97.44 | 25134956 | 65.02 | 24551502 | 63.51 | 6692374 | 17.31 | 120.7718 | 66.15997 |
| A1 | 51516386 | 51469798 | 99.91 | 38953302 | 75.61 | 38006050 | 73.77 | 14500887 | 28.15 | 161.5208 | 78.04109 |
| A2 | 48482874 | 48439198 | 99.91 | 41965656 | 86.56 | 41047470 | 84.66 | 14688676 | 30.3 | 163.6692 | 76.83277 |
| B | 43624090 | 43493538 | 99.7 | 31940642 | 73.22 | 27652466 | 63.39 | 3184915 | 7.3 | 105.2994 | 93.93268 |
| B1 | 37813938 | 37782634 | 99.92 | 28971134 | 76.61 | 28297192 | 74.83 | 9612758 | 25.42 | 163.1367 | 85.65466 |
| B2 | 43592668 | 43525314 | 99.85 | 39025718 | 89.52 | 38006884 | 87.19 | 8595654 | 19.72 | 134.11 | 80.6456 |
| C | 39024038 | 33989890 | 87.1 | 10162148 | 26.04 | 9705344 | 24.87 | 1480679 | 3.79 | 76.57509 | 38.83599 |
| C1 | 66775832 | 65504704 | 98.1 | 46669388 | 69.89 | 45343450 | 67.9 | 17460322 | 26.15 | 198.6205 | 146.8179 |
| C2 | 48714776 | 48320412 | 99.19 | 27757912 | 56.98 | 26861278 | 55.14 | 8141160 | 16.71 | 132.0523 | 82.97666 |
| D | 47682832 | 47558646 | 99.74 | 41979488 | 88.04 | 40234522 | 84.38 | 12620974 | 26.47 | 156.6184 | 90.10867 |
| D1 | 42113266 | 42070832 | 99.9 | 39097284 | 92.84 | 38145752 | 90.58 | 12995329 | 30.86 | 171.3483 | 82.34863 |
| D2 | 43945760 | 43838634 | 99.76 | 36448036 | 82.94 | 35321462 | 80.38 | 10622086 | 24.17 | 140.8983 | 72.13522 |

**Table s3. Read number for reads per kilobase per millions mapped reads (RPKM) of samples from four cell types**

|  | Gene Expression | | | | Transcript Expression | | | |
| --- | --- | --- | --- | --- | --- | --- | --- | --- |
| Samples | RPKM  >=1000 | RPKM  >=100 | RPKM  >=10 | RPKM  >=0.3 | RPKM  >=1000 | RPKM  >=100 | RPKM  >=10 | RPKM  >=0.3 |
| A | 36 | 976 | 6013 | 13565 | 31 | 874 | 7009 | 22609 |
| A1 | 60 | 1080 | 5447 | 13261 | 61 | 1016 | 6402 | 22170 |
| A2 | 49 | 998 | 5893 | 13661 | 48 | 913 | 6801 | 23489 |
| B | 75 | 890 | 5394 | 19864 | 73 | 862 | 5912 | 27995 |
| B1 | 35 | 865 | 6775 | 14638 | 36 | 758 | 7605 | 25298 |
| B2 | 39 | 791 | 6798 | 17558 | 37 | 659 | 7520 | 29091 |
| C | 45 | 907 | 5453 | 12072 | 40 | 843 | 6420 | 17720 |
| C1 | 51 | 858 | 5714 | 12441 | 46 | 784 | 6630 | 21519 |
| C2 | 55 | 866 | 5717 | 12866 | 51 | 800 | 6568 | 21427 |
| D | 102 | 1246 | 5288 | 15267 | 96 | 1252 | 6114 | 24201 |
| D1 | 37 | 850 | 6976 | 15083 | 36 | 714 | 7779 | 26415 |
| D2 | 74 | 1195 | 5751 | 14947 | 67 | 1148 | 6676 | 24571 |
